# An adolescent with primary cutaneous follicle center lymphoma: a case report and literature review

**DOI:** 10.3389/fonc.2023.1273719

**Published:** 2023-11-01

**Authors:** Wen-Yan Niu, Xue-Shen Yan, Han Qiao, Yu-Jiao Sun, Hai-Yan Gu, Guang-Lun Li, Zhong-Guang Cui, Juan Du

**Affiliations:** ^1^Department of Hematology, The Affiliated Hospital of Qingdao University, Qingdao, China; ^2^Department of Pathology, The Affiliated Hospital of Qingdao University, Qingdao, China

**Keywords:** follicle center lymphoma, cutaneous lymphoma, B-cell lymphoma, adolescent, case report

## Abstract

Primary cutaneous follicle center lymphoma (PCFCL) differs from follicular lymphoma in biological behavior and molecular profile and is treated as a distinct entity, according to the 5th edition of the World Health Organization classification of hematolymphoid tumors. It is an uncommon cutaneous B-cell lymphoma that is considerably rare in children and adolescents. To date, only 13 cases of individuals younger than 20 years of age have been reported in the literature. The lack of relevant clinical epidemiological data in this population has hampered the investigation of its clinical and diagnostic aspects. Here we report the case of a 17-year-old male with PCFCL, who may be the first PCFCL patient under 20 years of age reported in China. He was admitted to the hospital with a solitary nodule on his face. After complete surgical excision, the patient’s facial mass was histologically identified as PCFCL. The patient’s prognosis was favorable, with no recurrence at 17 months of follow-up after the surgical resection. We present a case of an adolescent PCFCL patient and systematically review the literature with a view to increase the awareness of the disease and inform the diagnosis and treatment of this age group.

## 1 Introduction

Primary cutaneous follicle center lymphoma (PCFCL) is a rare indolent cutaneous B-cell lymphoma (PCBCL) derived from follicle center cells and accounts for less than 1% of B-cell lymphoma cases. It is confined to the skin and rarely involves the lymph nodes or other extra nodal organs (1). PCFCL is much rarer in adolescents, with only 13 cases of PCFCL in children and adolescents reported to date (**Table 1**) (2–10). In this article, we report the case of a 17-year-old boy with PCFCL who was treated in our hospital and systematically review the literature to better understand the clinical and pathological characteristics of PCFCL in teenagers.

**Table 1:** Patients with primary cutaneous follicle center lymphoma under 20 years of age as reported in the literature.

Age at onset of disease (years)	Sex	Lesion location	Immunohistochemistry	Treatment and outcomes	References
17	Female	Scalp	Bcl-6, Bcl-2, CD20, and CD30 positive and CD10 negative	Excision and electron beam radiation therapy; alive at 25 months	Amitay-Laish I et al. (2)
8	Male	Right frontal scalp	CD10, CD20, and CD21 positive, Bcl-2 and CD30 negative	Excision; alive at 48 months	Condarco T et al. (3)
16	Female	Posterior arm	CD20, Bcl-6, CD10 positive, BCL2, and CD30 negative	Unknown	Edmonds N et al. (4)
16	Male	Left nose and left nasolabial fold	CD20, CD79a, Bcl-6 positive and CD5, CD10, CD30, Bcl-2, and CD21 negative	6 cycles of chemotherapy; alive at 41 months	Ghislanzoni M et al. (5)
16	Male	Left parietal scalp	CD20, Bcl-6 positive, and Bcl-2 negative	Excision; alive	Sayej WN et al. (6)
20	Female	Unknown	Unknown	Unknown; alive and recurrent at 13 months	Fink-Puches R (7)
11	Male	Medial canthus of the right eye	CD20, BCL6, CD10, and BCL2 positive	Excision; alive at 24 months	D’Alessandro PR et al. (8)
16	Female	Forearm	CD20, BCL2 positive, CD30, CD43, and TDT negative	Unknown	Al Harbi SM et al. (9)
<20	Three male and two female patients	Head and neck	Unknown	Unknown; alive at 48 months	Bomze D et al. (10)

## 2 Case report

**Figure 1:**
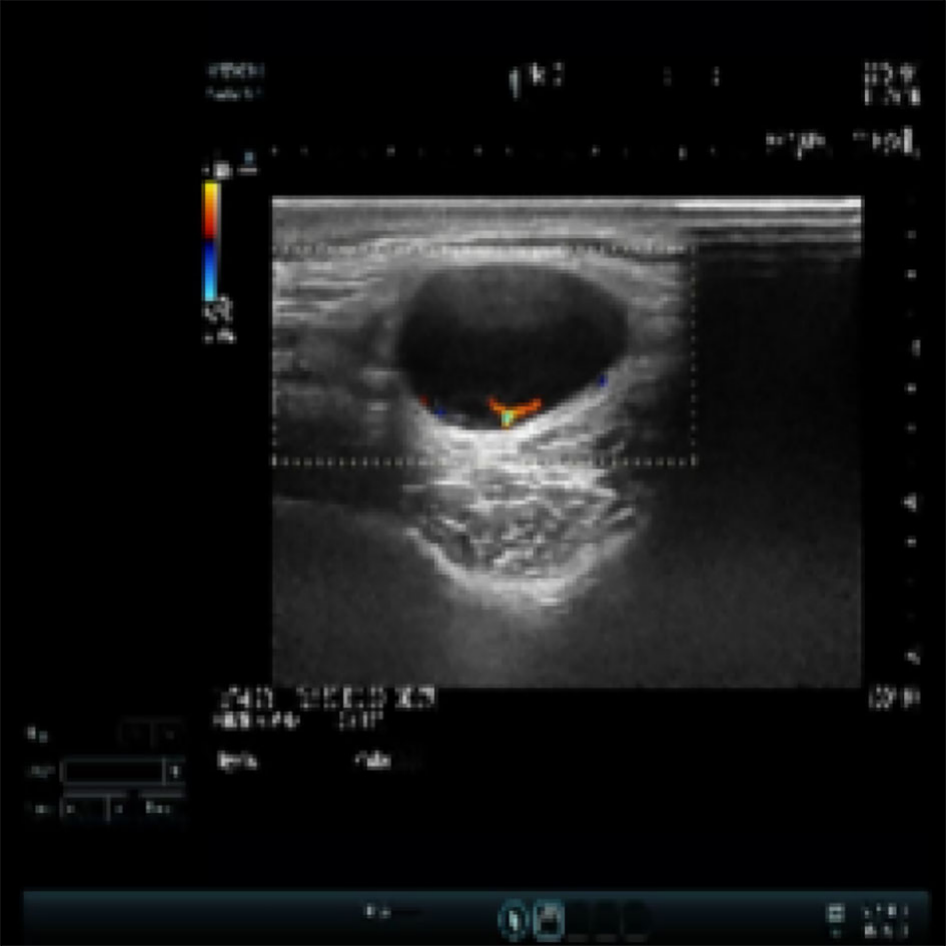
Body mass ultrasound of the patient.

**Figure 2:**
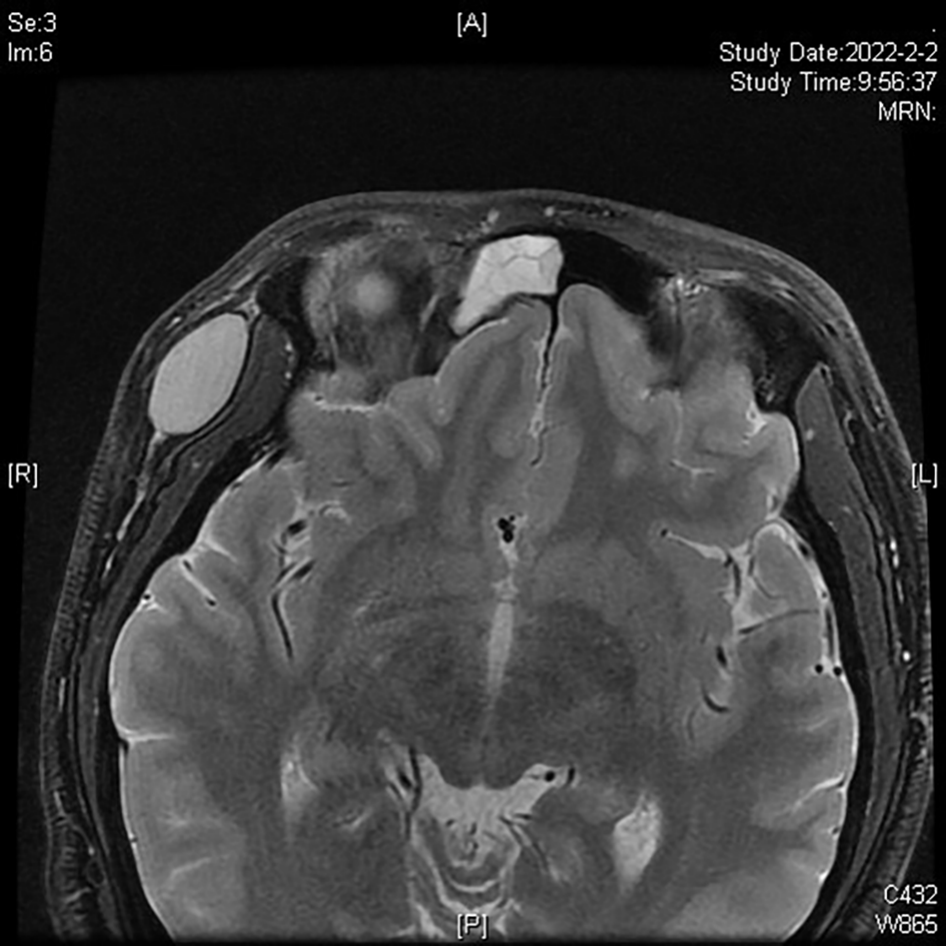
Orbital MRI showing a tumor in the right temporal area.

An Asian male aged 17 was admitted to our hospital in March 2022 due to “a facial nodule found for about 6 months.” A right temporal mass, initially the size of a peanut, was accidentally discovered in September 2021. The patient was previously healthy; the lump seemed to increase in size over time, and he visited our dermatology center in January 2022. An examination revealed a right temporal mass of approximately 2 cm in diameter; it was tough, with good mobility, clear borders, and a smooth surface. When the mass was imaged using ultrasound, a 2.2 cm ×1.9 cm ×1.1 cm very hypoechoic nodule with a regular shape, distinct borders, and uneven internal echogenicity was detected in the fatty layer of the right temporal region (**Figure 1**). An orbital MRI, performed in February 2022, showed an oval T1 and T2 compression lipid high-signal shadow with clear borders in the right temporal subcutis that was approximately 15 mm × 27 mm (**Figure 2**).

**Figure 3:**
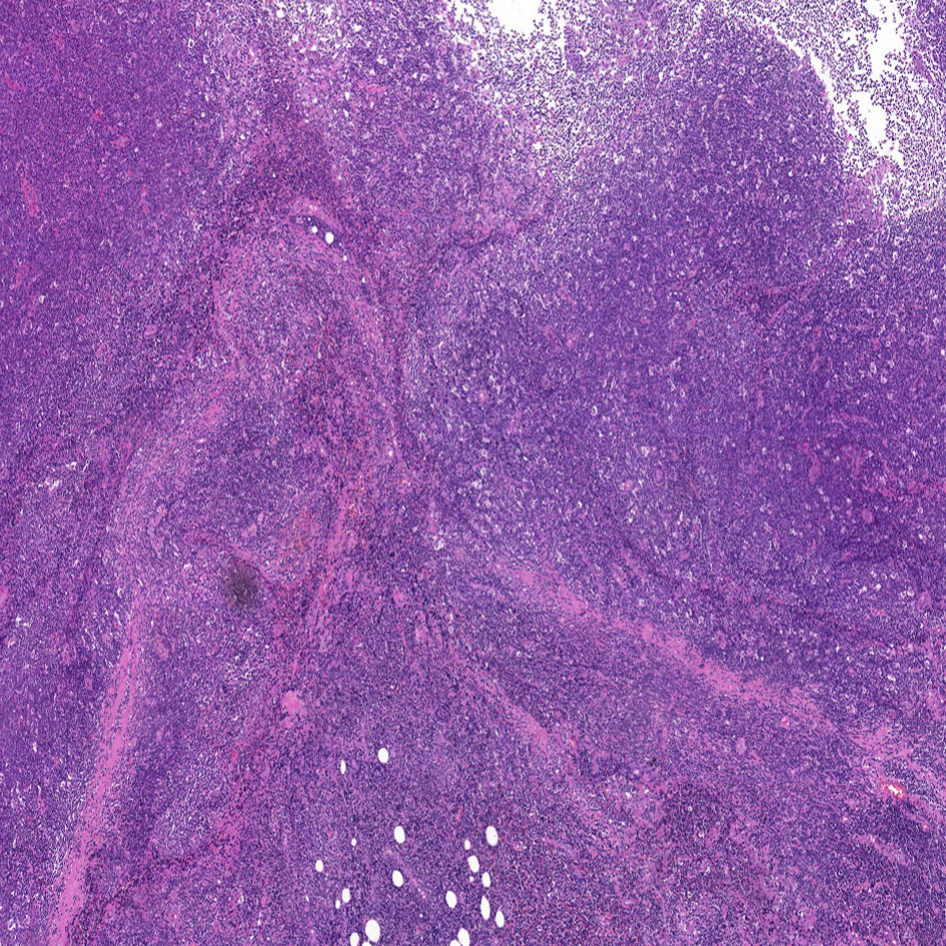
Hematoxylin and eosin staining of the tumor tissue (H&E, ×5). Neoplastic cells are small to moderately sized, with little cellular heterogeneity, and some infiltration of small lymphocytes and histiocytes can be observed in the background.

**Figure 4:**
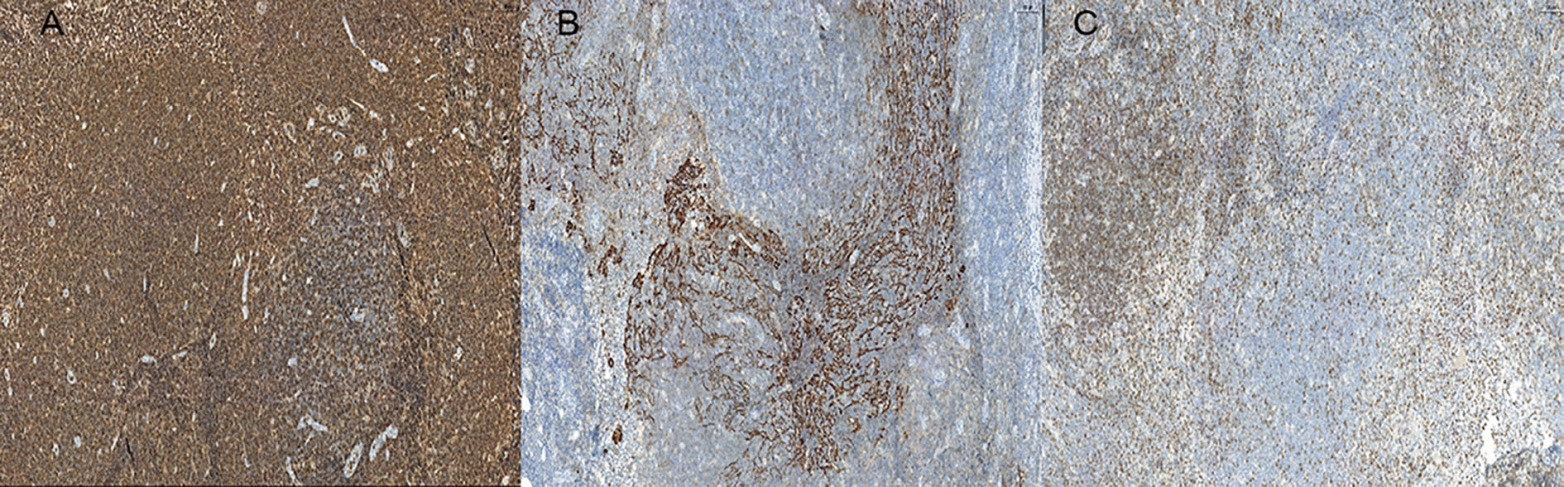
**(A)** Immunohistochemistry showing neoplastic cells diffusely positive for CD20 (×10). **(B)** Follicular dendritic cell networks strongly expressing CD21 (×10). **(C)** BCL-2 is variably expressed at different sites, and in this figure BCL-2 is negative (×10).

The patient was hospitalized in our department for oral and maxillofacial surgery on February 9, 2022. The ECOG score was 0. No significant abnormalities were observed on routine blood tests. The lactate dehydrogenase was 188.7 U/L (reference range, 120–250 U/L). It might be difficult to distinguish between benign and malignant skin cancers without a biopsy. On February 11, 2022, the patient underwent a right temporal mass resection and adjacent flap transfer repair under general anesthesia, during which the mass was completely removed, and the postoperative specimen was sent for pathological examination. The pathology showed multiple pieces of grayish-white disorganized tissue, totaling 2.3 cm ×1.8 cm ×0.7 cm. Hematoxylin and eosin (H&E) staining revealed lymphocyte infiltration with a follicular growth pattern. The follicular structure was atypical and lacked distinct mantle zones. Infiltrating cells comprised mainly follicular centrocytes and centroblasts, along with several histiocytes (**Figure 3**). The immunohistochemical staining showed a strong diffuse CD20 positivity for germinal center B-cells with PAX5 and PD1 positivity (**Figure 4A**). The follicular dendritic cell network was compressed with CD21-positive cells (**Figure 4B**). There was variability in Bcl-2 expression in the follicle center and interstitial areas (**Figure 4C**). In interstitial areas, there was positive Bcl-6 and Bcl-10 expression, and the ki67 proliferation index was 70%. The fluorescence *in situ* hybridization results suggested rearrangements of immunoglobulin heavy chains. Combined with the patient’s clinical condition (an isolated lesion with distinct borders) and the results of immunohistochemistry and Ig gene rearrangement tests, we considered a diagnosis of PCFCL.

The patient underwent enhanced CT of the neck, chest, and abdomen on March 28, 2022, which revealed no enlarged lymph nodes. The patient underwent bone marrow aspiration on March 28, 2022, and the bone marrow smear, flow cytology, and biopsy revealed no obvious abnormalities. The patient was diagnosed with PCFCL, and the TNM stage was T1N0M0. In view of the complete resection of the patient’s lesion, we advised that the patient be followed up and reviewed regularly. The patient cooperated well with regular telephone follow-ups. The patient underwent a cranial MR enhancement scan 45 days after surgery, which showed no significant abnormalities in brain MR enhancement and a postoperative change in the right temporal subcutaneous area. The patient was satisfied with the outcome of the procedure. At the time of submission, the patient had not relapsed during the telephone follow-up.

## 3 Discussion

In the 2018 World Health Organization for Research and Treatment of Cancer updated classification of cutaneous lymphomas, PCBCL has been divided into five categories: cutaneous marginal zone B-cell lymphoma (PCMZL), PCFCL, primary cutaneous diffuse large B-cell lymphoma, leg type (PCDLBCL-LT), intravascular large B-cell lymphoma, and EBV-positive mucocutaneous ulcer-provisional (11).

Clinically, PCFCL is more common in middle-aged men (>50 years old), with a preference for the head, neck, and back, and it typically presents as painless or pruritic. They can manifest morphologically as isolated or numerous plaques, nodules, papules, or masses (12). However, the incidence of PCFCL in adolescents differs significantly from that in adults. According to a retrospective study in the United States, which included 5,176 cases of cutaneous lymphoma, patients younger than 20 years of age accounted for only 10.4% of PCFCL cases, with an annual incidence rate of 0.12 per one million people. This represents an incidence rate of only one in 40 compared to the adult population (10).

According to histology, centroblast and follicle center cells make up the majority of tumor cells in PCFCL. Tumor cells show three growth patterns: follicular, diffuse, and follicular diffuse. Tumor cells were immunohistochemically tested positive for B-cell markers, such as CD20, CD79a, PAX5, and Bcl-6, but negative for CD5 and CD43 and mostly negative for MUM1. The expression of CD10 and bcl-2 varied, and their co-expression indicated a higher chance of skin recurrence (13). The strong expression of CD10 and bcl-2 suggests the possibility of systemic lymph node disease involving the skin, necessitating an appropriate differential diagnosis (14). Therefore, it is necessary to establish an appropriate differential diagnosis. PCMZL expresses CD20, CD79a, and bcl-2 but does not express CD10 and bcl-6 (15). The aggressive disease PCDLBCL-LT usually shows a strong expression of bcl-2 and MUM1. Unlike adult follicular lymphoma, most children and adolescents with PCFCL do not have the t (14, 16) chromosomal translocation involving BCL-2 protein expression.

Prior to staging assessment, it is essential to conduct a thorough medical history review, a comprehensive physical examination, blood routine tests, biochemistry tests (including lactate dehydrogenase), and either a CT or PET-CT scan. The necessity of bone marrow aspiration and biopsy remains a subject of debate. The TNMB staging system, originally designed for mycosis fungoides (MF) and Se’zary syndrome (SS), is not applicable to other primary cutaneous lymphomas. Consequently, the International Society for Cutaneous Lymphomas (ISCL) has recommended using an alternative TNMB staging system. A new TNM staging system was proposed by ISCL and EORTC for primary cutaneous lymphomas other than MF/SS (17, 18). The International Extranodal Lymphoma Study Group found that elevated lactate dehydrogenase levels, more than two skin lesions, and nodular lesions were independent risk factors affecting the prognosis of PCFCL; therefore, the international prognostic index (CIPI) for cutaneous lymphoma was based on these three risk factors to determine the prognosis of indolent cutaneous lymphoma. The CIPI for cutaneous lymphoma was developed based on these three risk factors. Patients with low-, intermediate-, and high-risk CIPI scores had 5-year progression-free survival rates of 91%, 64%, and 48%, respectively (*P* = 0.001) (16). In addition, differences in growth patterns, centroblast density, chromosomal abnormalities, and BCL-2 protein expression have no impact on the prognosis of PCFCL (12).

The preferred treatments are complete surgical resection or field radiation therapy, both of which have a complete remission rate of nearly 100% (19). In this case, there was no sign of recurrence in the short term after complete surgical resection. Rituximab alone or in combination with chemotherapy can be used to treat adult patients with extensive skin lesions (20). Radiotherapy alone has an increased rate of local recurrence compared with surgical resection (21). Delaying radiotherapy after recurrence does not affect prognosis (22). It is uncertain whether PCFCL in children and adolescents requires a different therapeutic approach than that in adults because of the rarity of this condition. In adult patients, PCFCL has a fair prognosis and an indolent course, with a 5-year survival rate of >95%. PCFCL is currently considered to have similar prognoses in pediatric and adolescent patients.

In conclusion, PCFCL is relatively rare in the pediatric and adolescent populations. The diagnosis of the disease is challenging due to the paucity of relevant studies and the lack of awareness of the disease, which may lead to underdiagnosis or misdiagnosis of the disease. Here we report a rare case of PCFCL in an adolescent in China and its multidisciplinary collaborative diagnostic and treatment process, which we hope will further raise awareness of the disease. In the future, as more prospective studies are conducted, the clinical and biological characteristics of PCFCL in pediatric and adolescent populations will be further elucidated.

## Data availability statement

The original contributions presented in the study are included in the article/supplementary material. Further inquiries can be directed to the corresponding authors.

## Ethics statement

The studies involving humans were approved by the ethics committee review board of The Affiliated Hospital of Qingdao University. The studies were conducted in accordance with the local legislation and institutional requirements. Written informed consent for participation in this study was provided by the participants’ legal guardians/next of kin. Written informed consent was obtained from the minor(s)’ legal guardian/next of kin for the publication of any potentially identifiable images or data included in this article.

## Author contributions

W-YN: Data curation, Writing – original draft, Writing – review & editing. JD: Supervision, Writing – review & editing. X-SY: Writing – original draft. HQ: Writing – original draft. Y-JS: Writing – original draft. H-YG: Resources, Visualization. G-LL: Supervision, Methodology. Z-GC: Supervision, Methodology.
